# Autophagic Machinery of Plant Peroxisomes

**DOI:** 10.3390/ijms20194754

**Published:** 2019-09-25

**Authors:** Sławomir Borek, Szymon Stefaniak, Jan Śliwiński, Małgorzata Garnczarska, Małgorzata Pietrowska-Borek

**Affiliations:** 1 Department of Plant Physiology, Faculty of Biology, Adam Mickiewicz University Poznań, Uniwersytetu Poznańskiego 6, 61-614 Poznań, Poland; szymon.stefaniak@amu.edu.pl (S.S.); jan.j.sliwinski@gmail.com (J.Ś.); garnczar@amu.edu.pl (M.G.); 2 School of Medicine, Medical Sciences and Nutrition, University of Aberdeen, Foresterhill, Aberdeen AB25 2ZD, UK; 3 Department of Biochemistry and Biotechnology, Faculty of Agronomy and Bioengineering, Poznań University of Life Sciences, Dojazd 11, 60-632 Poznań, Poland; malgorzata.pietrowska-borek@up.poznan.pl

**Keywords:** ATG proteins, macropexophagy, peroxins, pexophagy

## Abstract

Peroxisomes are cell organelles that play an important role in plants in many physiological and developmental processes. The plant peroxisomes harbor enzymes of the β-oxidation of fatty acids and the glyoxylate cycle; photorespiration; detoxification of reactive oxygen and nitrogen species; as well as biosynthesis of hormones and signal molecules. The function of peroxisomes in plant cells changes during plant growth and development. They are transformed from organelles involved in storage lipid breakdown during seed germination and seedling growth into leaf peroxisomes involved in photorespiration in green parts of the plant. Additionally, intensive oxidative metabolism of peroxisomes causes damage to their components. Therefore, unnecessary or damaged peroxisomes are degraded by selective autophagy, called pexophagy. This is an important element of the quality control system of peroxisomes in plant cells. Despite the fact that the mechanism of pexophagy has already been described for yeasts and mammals, the molecular mechanisms by which plant cells recognize peroxisomes that will be degraded via pexophagy still remain unclear. It seems that a plant-specific mechanism exists for the selective degradation of peroxisomes. In this review, we describe the physiological role of pexophagy in plant cells and the current hypotheses concerning the mechanism of plant pexophagy.

## 1. Introduction

Autophagy, or self-eating, is a conservative process that occurs in yeast, animal, and plant cells, and primarily involves the degradation of cytoplasmic fragments along with organelles, protein complexes, and other macromolecules. Autophagy in plant cells occurs at low intensity under normal conditions, but a clear enhancement of this process is observed as a result of various abiotic and biotic stresses (e.g., carbon, nitrogen, or phosphorus starvation, salinity, water deficit, high temperature, pathogen attack, reactive oxygen species) [1,2,3,4,5,6,7,8,9,10]. Under such circumstances, enhanced self-destruction delivers respiratory substrates and enables cell survival. In plants, autophagy participates in the circulation of cell components and acts as a quality control mechanism. It also functions in some developmental processes, such as pollen maturation [11], accumulation of storage protein in seeds [12], senescence [9], and cell death, including programmed cell death [2,13,14,15]. Autophagy coordinates the phytohormones to regulate plant growth and development under variable environments [16].

Despite the fact that autophagy has been known since the 1960s, it was thought until recently that this is a process during which various cell components are degraded in a non-selective manner (bulk autophagy). However, studies from recent years have provided a lot of physiological and molecular information indicating the functioning of selective kinds of autophagy [4,6,15,17,18,19,20,21]. Originally, selective kinds of autophagy were found in yeasts and animals. In contrast, selective kinds of autophagy in plant cells have been described just in the last few years. The first data on selective autophagy in plant cells are from 2006 and concern the degradation of protein complexes [22], and the first evidence for the occurrence of autophagic degradation of peroxisomes (pexophagy) in plant cells appeared in the literature at the turn of 2013 and 2014 [23,24,25,26]. In this review, we describe the physiological role of pexophagy in plant cells and the current hypotheses concerning the mechanism of plant pexophagy.

## 2. Macroautophagy

**Figure 1:**
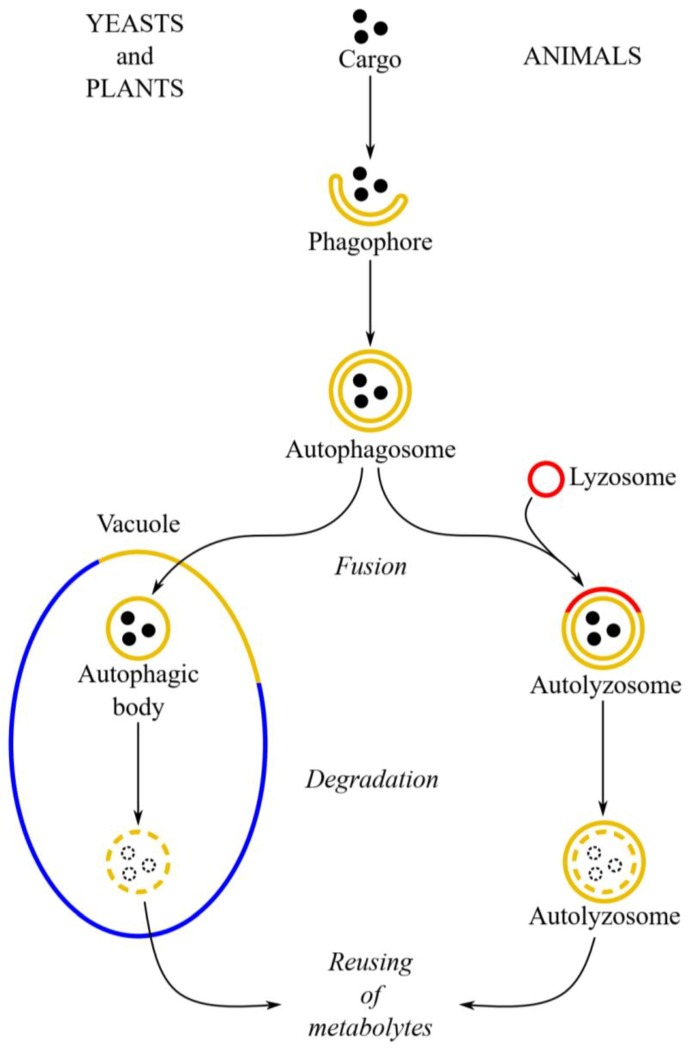
Schematic diagram depicting macroautophagy in yeast and plant cells (**left-hand side**) and in animal cells (**right-hand side**). In yeast and plant cells, the autophagosome fuses to the tonoplast, creating the autophagic body inside the vacuole. The autophagic body is degraded by hydrolases, allowing reuse of metabolites. In animal cells, the autophagosome fuses with the lysosome, giving the autolysosome where the cargo (autophagic body) is hydrolyzed. The degradation of peroxisomes via macroautophagy has been confirmed in yeast, plant, and animal cells.

The most investigated type of bulk and selective autophagy is macroautophagy. It begins with the appearance of an elongated vesicle (called a phagophore), which undergoes elongation, surrounding the cytoplasmic fragment together with the object to be degraded (Figure 1). It becomes an autophagosome, i.e., a fully developed vesicle with a double lipid–protein membrane, which contains a cargo targeted for degradation. The mature autophagosome in yeast and in plants fuses with the vacuole through the integration of the outer membrane of the autophagosome with the tonoplast [1,6,9,19,20,27,28,29,30,31]. In animals, the autophagosome fuses with the lysosome, creating an autolysosome [19,32,33,34]. Inside the vacuole or autolysosome, the internal membrane of the autophagosome with the cargo forms an autophagic body, which is rapidly degraded by the action of lytic enzymes [1,19,20,27,28,30,33,35,36]. During macroautophagy, autophagy-related proteins (Atg) play the pivotal role [1,2,6,9,20,27,37,38]. So far 42 Atg proteins have been described [39]. *ATG* genes are mostly conservative in yeasts, animals, and plants [37,40]. About 16–18 *ATG* genes are common to different kinds of autophagy, including bulk and selective autophagy. Products of these genes are involved in the formation of autophagosome and are therefore referred to as core proteins for autophagy [2,9,20,27,37]. One of the best-known proteins involved in autophagy in yeasts and plants is Atg8, which plays an important role in the formation of the autophagosome [6,17,20,28,30,35,37,41]. Atg8 is attached to the growing phagophore, both to the inner and outer membrane. Phosphatidylethanolamine is the Atg8-binding site [20,29,35,41,42]. After binding of Atg8 to the phagophore, this protein interacts with various receptors and adapters (including other Atg proteins) enabling the recognition of cell organelles intended for autophagic degradation [17,28,35,37]. The animal homolog of the Atg8 protein is microtubule-associated protein-1 light chain 3 (LC3). This protein is involved in the functioning of microtubules and occurs in all types of tissues. The formation of the phagophore induces conversion of LC3-I into LC3-II, which involves binding of phosphatidylethanolamine. LC3-II is located in the membrane of the autophagosome and ensures its structural stability [19,43].

## 3. Microautophagy

**Figure 2:**
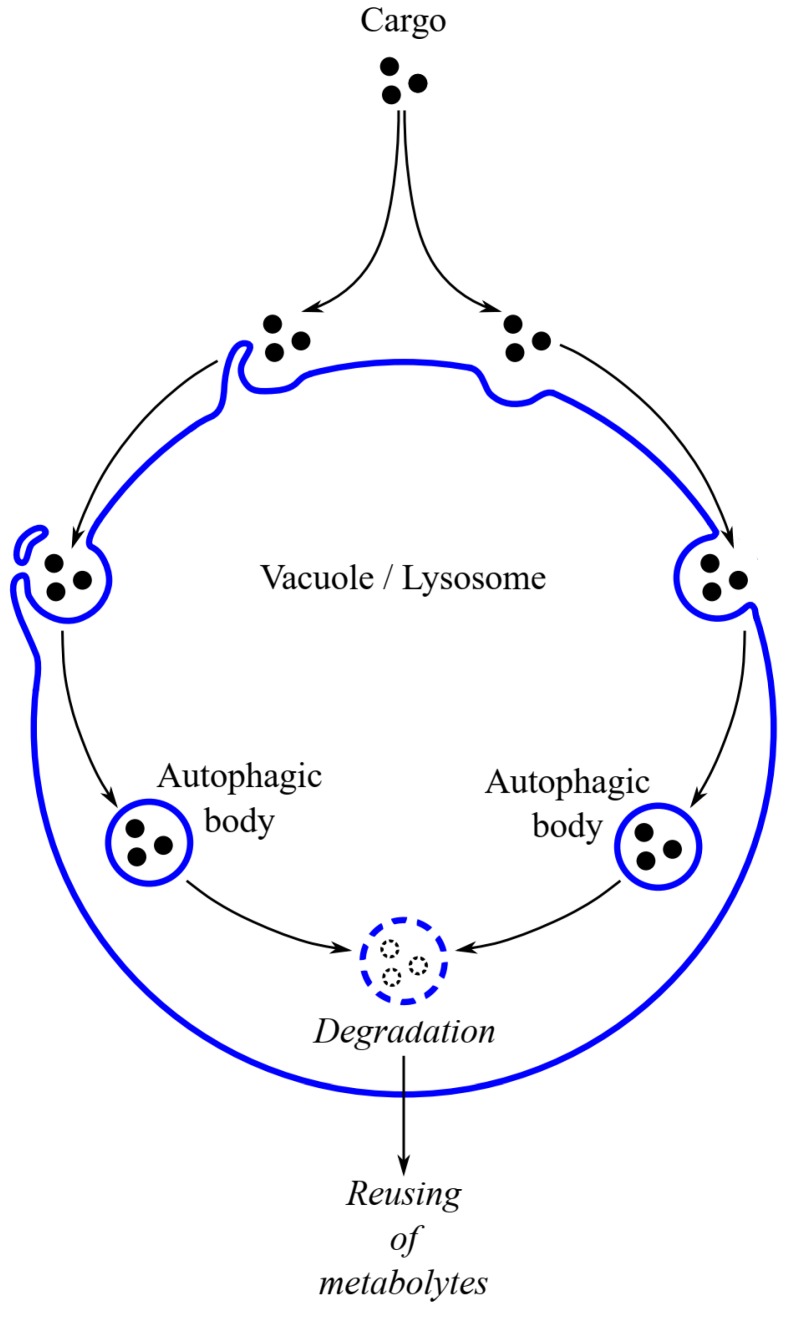
Schematic diagram depicting microautophagy in eukaryotic cells. The general scheme of microautophagy in yeast, plant, and animal cells is very similar. During this kind of autophagy, an autophagosome is not created, and the cargo is engulfed into the vacuole or lysosome directly from the cytosol. A protrusion or invagination may be formed from the vacuolar or lysosomal membrane, sequestering the cargo into the lumen of the lytic compartment of the cell. The degradation of peroxisomes via microautophagy has been confirmed only in yeast cells.

During this process, an autophagosome is not formed, but a fragment of the cytoplasm with organelles is absorbed into the vacuole or lysosome by invagination or protrusion of vacuolar or lysosomal membrane (Figure 2) [44]. During micropexophagy in yeast, a flat autophagic membrane structure (called the micropexophagy-specific apparatus, MIPA) emerges on the peroxisome surface and fuses with a protrusion of the vacuolar membrane, thereby engulfing the peroxisomes. In the lumen of the vacuole or lysosome, a single-membrane vesicle is formed, which is called an autophagic body [30,35,45,46,47,48]. Microautophagy seems to proceed in a similar way in yeast, animals, and plant cells. However, the amount of literature data on microautophagy is considerably smaller than the data concerning macroautophagy.

## 4. Pexophagy: Selective Degradation of Peroxisomes

Peroxisomes are small single-membrane bound organelles occurring in all eukaryotic cells [49,50,51]. In plants, these organelles participate, among others, in storage lipid mobilization during seed germination and seedling development. In these organelles there occur β-oxidation of fatty acid and the glyoxylate cycle, which convert fatty acids to succinate and malate [52,53,54]. Peroxisomes are also involved in photorespiration and the synthesis of phytohormones, which are critical for signaling pathways, including jasmonic acid, auxin, and salicylic acid [51,54]. Peroxisomes are an important component of the antioxidant system in cells, harboring enzymes involved in reactive oxygen and nitrogen species detoxification. One of them is catalase, the key enzyme in the detoxification of hydrogen peroxide, which is generated in peroxisomes in greater amounts than in other subcellular compartments [50,53,54,55]. Peroxisomes are extremely dynamic organelles, because their number and function may change considerably due to changes in the environment, both intra- and extracellular [51,53,56,57]. For example, germinating seeds, which depend on fatty acid β-oxidation and the glyoxylate cycle to use stored lipid, develop peroxisomes that are enriched in glyoxylate cycle enzymes such as isocitrate lyase and malate synthase. Such peroxisomes are called glyoxysomes. However, during seedling development and storage lipid depletion, isocitrate lyase and malate synthase are eliminated from peroxisomes in favor of enzymes required for photorespiration [49,58].

Initially, pexophagy was best studied in methylotrophic yeasts (*Pichia pastoris*, *Hansenula polymorpha*, or *Saccharomyces cerevisiae*) that can feed on methyl alcohol [40,47,59,60,61]. Due to the easy induction of peroxisome proliferation in methylotrophic yeasts, these organisms constitute an excellent research model for analysis of the course of pexophagy. By adding methyl alcohol or oleic acid to the growth medium, the number of peroxisomes in the cells can be easily increased. A decrease in the number of peroxisomes can also be induced equally easily. This is possible due to the elimination of a proliferating factor from the medium [59,60,61,62]. The methyl alcohol or oleic acid can be replaced with another compound, being a carbon source in the medium, e.g., with glucose or ethyl alcohol. Under natural conditions, the change in the number of peroxisomes is a sign of adaptation of yeast to environmental conditions [59,61]. In mammals, pexophagy is essential for the maintenance of homeostasis of peroxisomes, which is necessary for the prevention of various peroxisome-related disorders [63,64], and the study of pexophagy is possible after peroxisome proliferation obtained through the use of appropriate medicaments [64]. Research on pexophagy in plant cells has not been performed until recently, because unlike in yeast and mammalian cells, it is very difficult to induce peroxisome pre-proliferation in plant cells to later examine their degradation. In plants, pexophagy contributes to peroxisome homeostasis by degrading damaged or obsolete peroxisomes [57,65]. Moreover, pexophagy seems to occur at a higher basal rate than selective autophagy of other organelles, as evidenced by the higher relative accumulation of peroxisomal proteins compared to proteins of other organelles such as Golgi, endoplasmic reticulum, mitochondrion, and chloroplast in the *Arabidopsis thaliana* autophagy-defective mutant *atg5* [25,26].

### 4.1. Micro- and Macropexophagy

There are two main models of pexophagy in yeast, i.e., macropexophagy and micropexophagy [27,40,47,59,61,62,66,67]. Micropexophagy is much slower than macropexophagy and the course of micropexophagy depends on newly synthesized proteins, e.g., functional proteases (acting inside the vacuole) and functional phosphatidylinositol 3-kinase [62]. During micropexophagy in yeasts the micropexophagy-specific apparatus (MIPA) appears, which is essential for absorbance of peroxisomes by the vacuole [19,44,47,48,59,60,66,67,68,69]. The initiation of micropexophagy or macropexophagy in yeast cells may be determined by the carbon source as well as the appropriate level of ATP in the cell. In *Pichia pastoris*, an elevated level of ATP induces micropexophagy, while the reduced content of ATP stimulates macropexophagy. Due to the necessity of tonoplast reorganization during micropexophagy, it is suggested that this process requires more energy than macropexophagy [61]. In *Pichia pastoris*, the ATP level is a much more important factor in the induction of pexophagy than the type of carbon source used for growth. In addition, it was found that under the high level of ATP in cells of this fungus there is a tendency to degrade all peroxisomes, regardless of their condition [59]. Although pexophagy has already been confirmed in plant cells [23,24,25,26] and despite the clear evidence of micropexophagy in yeast, there is still no evidence of micropexophagy in plant cells. Until now, only macropexophagy has been observed in plant cells.

### 4.2. Developmentally and LON2 Protease/Chaperone Dysfunction-Induced Pexophagy

The occurrence of selective, autophagic degradation of peroxisomes for their recycling is indispensable for the proper development of plant organisms [26], from the germination phase of seeds to the maintenance of homeostasis in mature individuals. Degradation of peroxisomes or some proteins in peroxisomes is very important because during seed germination and seedling growth peroxisomes, also called glyoxysomes, participate in the breakdown of storage lipid (β-oxidation of fatty acids and the glyoxylate cycle), while in the photosynthetic tissues of older plants they are involved in photorespiration. Such peroxisomes are then called leaf peroxisomes. Under such circumstances, some enzymes are no longer needed, and they must be degraded [49]. Experiments performed on *Arabidopsis thaliana atg5-1* and *atg7-2* mutants showed increased accumulation of CFP-SKL (a peroxisomal marker) in hypocotyls of developing seedlings in comparison to wild-type seedlings, and additionally, this peroxisomal marker was localized in the vacuole of wild-type hypocotyls but not in vacuoles of the *atg7-2* mutant. This observation suggested a role of autophagy in the degradation of peroxisomal proteins. It was confirmed for isocitrate lyase and malate synthase, two marker enzymes of the glyoxylate cycle. The degradation of these two enzymes in hypocotyls of *atg* mutants was clearly delayed compared to the degradation in the wild-type seedlings. However, the inhibition of isocitrate lyase and malate synthase degradation in the hypocotyls of mutants was only transient, suggesting the occurrence of degrading processes other than autophagy, for example proteolytic degradation by peroxisomal matrix proteases [24]. Pexophagy in plants is also tissue-dependent. In *Arabidopsis thaliana*, pexophagy was observed in the hypocotyl [24] and leaves [26] but not in the cotyledons [24] and roots [26].

During the functional transition of glyoxysomes into leaf peroxisomes, obsolete enzymes are degraded inside peroxisomes by LON2 protease, while newly synthesized enzymes are transported into the peroxisome [49]. LON2 protease is a peripheral membrane protein, which associates with the membrane from the matrix side [70] and degrades various peroxisomal proteins, including isocitrate lyase, malate synthase (glyoxylate cycle), and thiolase (β-oxidation of fatty acids) [23,49,70]. It has been shown that in *Arabidopsis thaliana* peroxisomes with nonfunctional LON2 protease undergo pexophagy. In wild-type plants, when peroxisomes during plant development change their function, isocitrate lyase and malate synthase disappeared and the content of thiolase was significantly reduced. In the *lon2* mutant lacking a functional LON2 protease, it was found that the above-mentioned three enzymes also disappeared, not however due to the LON2 protease activity, but whole peroxisomes were degraded by autophagy. However, in the *lon2atg* double mutant, in which autophagy does not occur, the above-mentioned enzymes were maintained at a constant level. These data suggest that LON2 protease facilitates matrix protein degradation during peroxisome content remodeling, provide evidence for the existence of pexophagy in plant cells, and indicates that peroxisome destruction via autophagy is enhanced when LON2 is absent [23]. The *Arabidopsis thaliana apem10* mutant (impaired in LON2 activity) displayed enlargement of peroxisome size, as well as acceleration of peroxisome degradation, leading to a considerably reduced number of peroxisomes. In the *apem10/peup1* double mutant (*peroxisome unusual positioning 1*), in which peroxisome degradation is arrested due to the defect of ATG2, the peroxisome number increased compared with *apem10*. Additionally, the aggregation of peroxisomes was frequently observed in *apem10/peup1* [70]. Peroxisome aggregation is typical for *peup1* and is caused by oxidative damage of peroxisomal matrix proteins [25]. These results demonstrate that the decrease in peroxisome number is caused by accelerated peroxisome degradation via autophagy [70]. Analyzing the content of isocitrate lyase, malate synthase, and thiolase the authors of this study concluded that glyoxysomal proteins are degraded by two independent pathways. One is the LON2-dependent, and the second is the autophagy-dependent pathway. In contrast to isocitrate lyase and malate synthase, thiolase was not degraded in the *peup1* mutant, indicating that thiolase is not a substrate of LON2 protease, but it is degraded by autophagy during the peroxisomal functional transition [70]. It was also shown that LON2 protein acts as a chaperone. The chaperone function of LON2 suppresses peroxisome degradation by autophagy, but the proteolytic function interferes with the suppression, indicating that modulation between the proteolytic and chaperone functions of LON2 regulates the degradation of peroxisomes by autophagy [70]. If the chaperone function of LON2 can suppress pexophagy, it suggests that misfolded or aggregated matrix proteins may be signals for pexophagy. How peroxisomes with absent or impaired LON2 protease/chaperone are marked for autophagic turnover is not yet clear in plants, but such a signal would presumably traverse the peroxisome membrane to be recognized by a cytosolic pexophagy machinery [20].

### 4.3. Sugar Starvation-Induced Pexophagy

Pexophagy has been observed in the suspension of BY-2 tobacco (*Nicotiana tabacum*) cells. It was found that a significant peroxisome pool undergoes autophagic degradation under sucrose starvation conditions, and the accumulation of peroxisomes was observed in cells treated with the autophagy inhibitor 3-methyladenine. Additionally, 3-methyladenine caused an increase in peroxisomal proteins and cellular peroxisome numbers in rapidly dividing tobacco cells under nutrient-rich conditions. These data demonstrate that a large fraction of the peroxisome pool is subject to extensive autophagy-mediated turnover under both nutrient starvation and optimal growth conditions [71]. It is also assumed that pexophagy may occur in cells of sugar-starved embryonic axes of germinating seeds of various species of lupin (*Lupinus*) [52,72,73]. The main storage compound in lupin seeds is protein, but these seeds may also contain a large amount of lipid (up to 50% of seed dry matter [74]), in the decomposition of which the peroxisomes are involved during seed germination. Under artificially induced sugar starvation, the embryonic axes of lupin contain much more lipid than axes fed with sucrose. This indicates the limitation in lipid breakdown under carbon starvation conditions [72,74]. At the same time, the observations of the ultrastructure of the cells of these organs showed that the autophagic bodies inside the vacuole contain organelles, which may be more or less uniquely identified as peroxisomes [73].

### 4.4. Oxidative Damage-Induced Pexophagy

Peroxisomes have intensive oxidative/antioxidative metabolism. Reactive oxygen species (ROS) are generated in these organelles and simultaneously they are detoxified by antioxidant enzymes such as catalase [50,53,54,55]. Such intensive oxidative/antioxidative metabolism causes the peroxisomal proteins to undergo oxidative damage, and they must be continuously removed from peroxisomes. ROS accumulation and oxidative damage require the whole peroxisomes to be degraded via pexophagy, which in plant cells is a part of the peroxisome quality control system [18,25,26,75,76]. In *Arabidopsis thaliana peup1*, *peup2*, and *peup4* mutants can increase in the number of peroxisomes and a tendency of the peroxisome to form aggregates has been observed. It was also found that the above-mentioned mutants are identical to the *Arabidopsis thaliana* mutants with impaired autophagy (*atg2*, *atg18a*, and *atg7*, respectively). The increase in the number of peroxisomes in the peup1 mutant clearly proves that the autophagic degradation of these organelles is impaired. At the same time, aggregated peroxisomes showed an increase in the inactive catalase content, which caused the peroxisomes in the mutants to contain more ROS, and their components were significantly more oxidized than in the wild-type plants. Similar aggregation of peroxisomes can also be induced in wild-type plants by exogenous application of H_2_O_2_. Aggregated peroxisomes also appeared in the *Arabidopsis thaliana* mutant cat2 with decreased catalase activity [25]. There was also observed (using fluorescence techniques) frequent coexistence of aggregated peroxisomes and ATG8 (a marker of autophagosomes). The above-mentioned data clearly prove that the peroxisomes damaged by H_2_O_2_ are selectively degraded by autophagy in plant cells [25]. A team of other researchers [26] also came to identical conclusions, performing research on a different set of *Arabidopsis thaliana* mutants (*atg2*, *atg5*, *atg7*, and *atg9*). Considering that peroxisomes with components damaged by ROS undergo pexophagy, it may be assumed that H_2_O_2_ generated inside the peroxisomes is the signal triggering the sequence of events leading to the utilization of these organelles in the vacuole [76]. Aggregation of peroxisomes was also observed in tobacco BY-2 suspension cells, where the activity of catalase was artificially inhibited by aminotriazole [77].

### 4.5. Peroxisome Receptor/Adaptor and Scaffold Proteins

**Figure 3:**
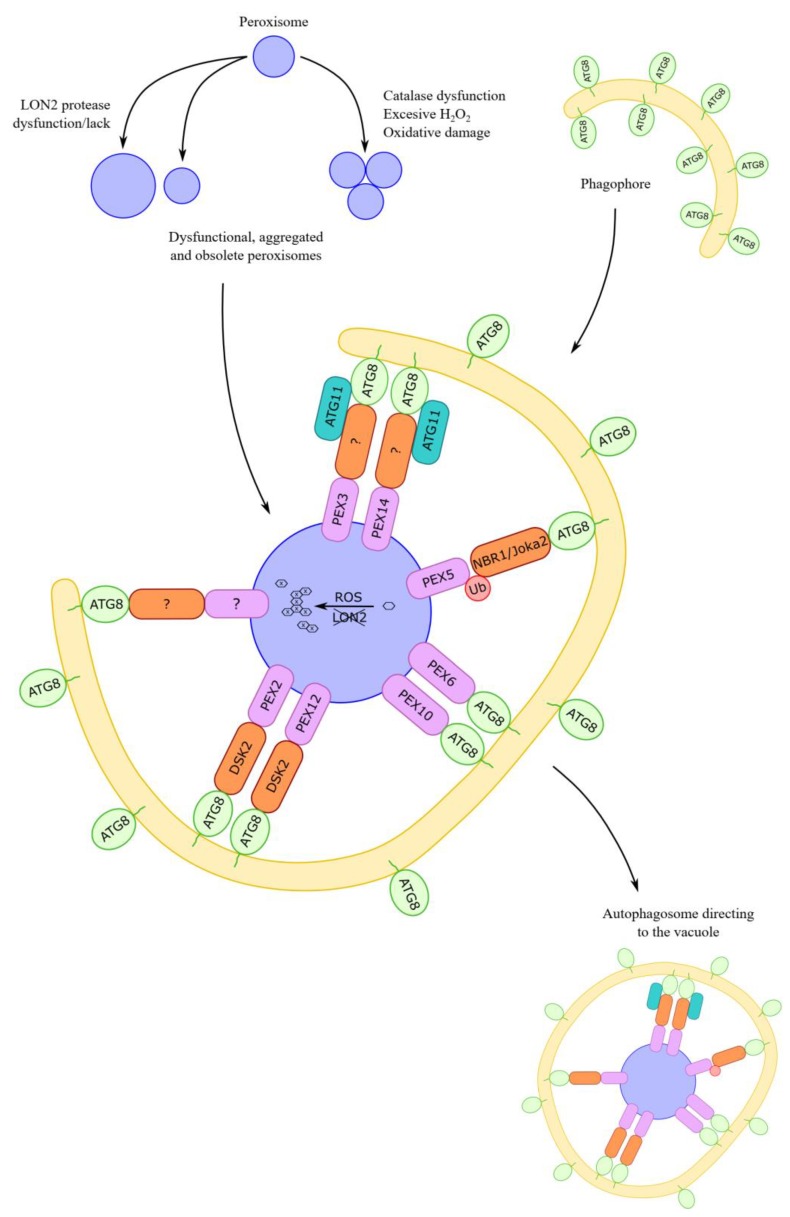
Hypothetical working model of macropexophagic apparatus in plant cells. For a detailed description see main text. The receptors/adaptors, as well as scaffold proteins, involved in the pexophagy in plant cells are still not known. It is postulated that plant NBR1, Joka2 (a hybrid homolog of mammalian NBR1 and p62), and DSK2 can play the role of receptor/adaptor during pexophagy. The signals coming from the surface or from the matrix of the plant peroxisome are also not known; however, based mainly on the data concerning yeasts and mammals, the following proteins are taken into consideration: peroxisomal membrane proteins PEX3 and PEX14, peroxisomal membrane proteins with the identified Atg8-interacting motif (AIM), PEX6 and PEX10, ubiquitinated PEX5, peroxisomal membrane proteins with RING-finger domain PEX2 and PEX12, and oxidized or aggregated matrix proteins. Additionally, ATG8 can be considered as a receptor that may first recognize the target, bind to peroxisomal membrane proteins by AIM, and then bind to phosphatidylethanolamine in the growing phagophore. Among scaffold proteins, only ATG11 is suggested to be involved in plant pexophagy, but it is not known which peroxisome receptor/adaptor it could interact with.

Although pexophagy has been evidenced in plant cells, little is known about receptor/adaptor proteins of peroxisomes targeted for pexophagy in plants. Additionally, little is known about other proteins recruited in autophagic machinery during plant pexophagy. To date, several hypotheses have been formulated, mainly based on knowledge concerning yeasts and mammals. Figure 3 shows the summarized data on macropexophagic machinery in plant cells.

In *Pichia pastoris*, such a receptor is Atg30 [68,78]. This protein is anchored in the peroxisome membrane through interactions with peroxins PEX3 and PEX14 [60,68]. Peroxins are a group of peroxisome biogenesis proteins, which are an important part of the peroxisomal import machinery. They are conserved from fungi, animals to plants, and some of them (RING domain-containing peroxins) act as E3 ligase [79]. Atg30 plays an important role in the formation of both the MIPA during micropexophagy and autophagosome during macropexophagy. It undergoes multiple phosphorylation and interacts with scaffold proteins Atg11, Atg17 [68], and Atg8 (decorating the phagophore/autophagosome membrane), which are necessary for the occurrence of pexophagy [78]. Another important protein in pexophagy in *Pichia pastoris* is Atg37, which regulates interactions between Atg30 and Atg11 [80]. In *Saccharomyces cerevisiae*, Atg36, whose peroxisome docking site is PEX3, is the marker protein for pexophagy. Atg36 also undergoes the necessary phosphorylation [78] and binds the scaffold proteins, including Atg11 and Atg8 [81]. In *Hansenula polymorpha*, only PEX14 plays an important role in pexophagy, as PEX3 is removed from peroxisomes and is not degraded by macropexophagy [60]. Details on pexophagy receptors/adaptors and scaffold proteins in yeast were recently reviewed by Gatica and coworkers [82] and Eberhart and Kovacs [83]. Atg30 and Atg36 proteins are specific only to fungal cells because their homologs have not been found in other organisms, either in mammals [64,82,83] or plants. However, an orthologue of Atg11 has been identified in *Arabidopsis thaliana* (AtATG11). This plant orthologue binds Atg8 via the ATG8-family-interacting motif (AIM) and plays a role in mitophagy and bulk autophagy during senescence [84]; however, there is no evidence that AtATG11 is involved in plant pexophagy.

In mammals, the protein neighbor of BRCA1 Gene 1 (NBR1), which attaches to the ubiquitinated PEX5, is the peroxisomal receptor/adaptor for pexophagy [85,86] or peroxisome membrane protein PMP70 [86]. Another receptor/adaptor that may be involved in pexophagy in animals is p62 (SQSTM1/Sequestome 1) binding ubiquitinated peroxisomes [87]. p62 is not required when NBR1 is in excess, but its binding to NBR1 increases the efficiency of NBR1-mediated pexophagy [85]. PEX5 and PMP70 are ubiquitinated by E3 ubiquitin ligase peroxin 2 (PEX2). Expression of PEX2 leads to gross ubiquitination of peroxisomes and degradation of peroxisomes via pexophagy during amino acid starvation [86]. NBR1 and p62 are also receptors/adaptors for other components of mammalian cells undergoing selective autophagic degradation because they can attach to various ubiquitinated proteins [82,88,89]. Both NBR1 and p62 also bind LC3, which decorates the phagophore/autophagosome membrane in mammals. Details on pexophagy receptors/adaptors and scaffold proteins in mammalian cells were recently reviewed by Zientara-Rytter and Subramani [90], Cho and coworkers [64], Gatica and coworkers [82], and Eberhart and Kovacs [82]. In plants, the NBR1 homolog was identified in *Arabidopsis thaliana*. NBR1 is accumulated in *Arabidopsis thaliana* mutants lacking autophagy [91] and recognizes ubiquitinated cargo. Plant NBR1 is implicated in autophagic degradation of ubiquitinated protein aggregates during heat stress [92,93] and in limiting viral infection by targeting cauliflower mosaic virus capsid proteins [94] and turnip mosaic virus silencing the suppressor HCpro [95] for autophagic degradation. However, NBR1 is not involved in autophagic degradation of proteasomes in *Arabidopsis thaliana* [96]. In plants, Joka2 protein was also found. This is a hybrid homolog of animal NBR1 and p62, which may be involved in selective autophagy in tobacco BY-2 suspension cells [77,97,98]. Nevertheless, there is no clear evidence that NBR1 and Joka2 are peroxisome receptors/adaptors during plant pexophagy. It is rather probable that NBR1 is not necessary for pexophagy in plant cells because it was shown that overexpression of NBR1 in the *Arabidopsis thaliana lon2* mutant is not sufficient to trigger autophagy of seedling peroxisomes, indicating that in this plant species an NBR1-independent mechanism to target peroxisomes for autophagic degradation exists [99].

It is suggested that a marker of peroxisomes targeted for pexophagy in plant cells may be ATG8, because the coexistence of ATG8 and peroxisomes, particularly aggregated peroxisomes, has been observed in *Arabidopsis thaliana* [24,25,26]. This finding supports the model of selective autophagy, in which ATG8 first recognizes a target by direct interaction with an ATG8-family-interacting motif (AIM) and then is conjugated to phosphatidylethanolamine to recruit and expand the phagophore in the proximity of the target [26]. Such data constitute strong grounds for recognizing ATG8 as a marker of peroxisome, which is targeted for autophagic degradation in plant cells, but it is not known to which peroxisomal protein (or proteins) ATG8 can bind [24,25,26,75]. However, it is known that most of the proteins that are specifically turned over by selective autophagy are recognized by the presence of short AIMs that facilitate their association with the autophagy apparatus. Applying bioinformatic methods it was possible to predict in silico AIMs in AIM-containing proteins on a genome-wide scale in various organisms. Such analysis identified nine peroxisomal PEX proteins in *Arabidopsis thaliana* that contain AIMs, among which AtPEX1, AtPEX6, and AtPEX10 possess evolutionarily conserved AIMs. Bimolecular fluorescence complementation results verified that AtPEX6 and AtPEX10 indeed interact with ATG8 in plants, suggesting that they could drive pexophagy [100]. If ATG8 would bind directly to peroxisomal membrane proteins, a bridging receptor/adaptor would not be necessary.

The ubiquitin-binding protein DSK2 (dominant suppressor of KAR2) is another pexophagy receptor/adaptor candidate in plants. DSK2 is a member of the ubiquitin receptor family known to function as shuttle factors ferrying polyubiquitinated substrates to the proteasome for degradation [79]. *Arabidopsis thaliana* DSK2 binds to the transcription factor BES1 (BRI1-EMS Suppressor 1), which functions as a master regulator in the brassinosteroid pathway that promotes plant growth. BES1 interacts with the ubiquitin receptor protein DSK2 and is targeted to the autophagy pathway during stress via the interaction of DSK2 with ATG8 through AIMs [101]. DSK2 also interacts with two peroxisomal membrane proteins, PEX2 and PEX12, through the RING (really interesting new gene) finger domain. These results suggest that *Arabidopsis thaliana* peroxins containing the RING domain can act together with DSK2 in the peroxisomal membrane-associated protein degradation system [79]. However, the involvement of DSK2 in plant pexophagy has not been confirmed yet.

## 5. Conclusion and Future Perspectives

The results of studies performed in recent years clearly indicate that the view on autophagy had to change. Autophagy not only makes it possible for the cell to survive in conditions of carbon or nitrogen starvation, but it is also an extremely important process involved in the metabolic turnover, in maintaining cell homeostasis, and in defense reactions. These functions of autophagy, however, are associated with very precise and selective degradation of particular cell components. While the general scheme of the course of autophagic degradation of organelles, protein complexes, or macromolecules is already quite well known, many questions remain unanswered. An example is the relatively modest knowledge of the receptors of particular cellular components and the way they are marked for autophagic degradation. For many years, it was thought that autophagy is a conservative process and occurs very similarly among different, unrelated groups of organisms. Often, knowledge about autophagy concerning, for example, yeast has been extrapolated to mammalian or plant cells. An example is the assumption, originating many years ago, that pexophagy occurs in plant cells. Although this process has already been confirmed in plants, it turns out that, for example, receptors or adapters for plant peroxisomes are different than those in yeast or mammal cells. Therefore, current and future research on the initial stages of pexophagy seem to be extremely important for the detailed understanding of this process in plant cells. Future studies should reveal whether PEX proteins are responsible for pexophagy in plant cells and if NBR1 also plays a role in peroxisome recycling similar to mammalian cells. It was evidenced that elevated H_2_O_2_ levels and LON2 protease dysfunction cause accumulation of nonfunctional proteins inside the peroxisome, but nothing is known about how the endogenous signal is transferred to the surface of the peroxisome and which protein or proteins are responsible for docking of the autophagic machinery to the peroxisome. Another question still to be answered is whether micropexophagy occurs in plant cells.

## Abbreviations

AIM	ATG8-family-interacting motif
ATG	Autophagy-related gene
BES1	BRI1-EMS suppressor 1 transcription factor
DSK2	Dominant suppressor of KAR2
Joka2	Hybrid homolog of animal NBR1 and p62
LC3	Microtubule-associated protein-1 light chain 3
LON2	LON protease/chaperone 2
MIPA	Micropexophagy-specific apparatus
NBR1	Neighbor of BRCA1 gene 1
p62	Sequestome 1
PEX	Peroxin
PMP	Peroxisome membrane protein
RING	Really interesting new gene
ROS	Reactive oxygen species
SQSTM1	Sequestome 1
